# Dual functional states of working memory realized by memristor-based neural network

**DOI:** 10.3389/fnins.2023.1192993

**Published:** 2023-06-07

**Authors:** Hongzhe Wang, Xinqiang Pan, Junjie Wang, Mingyuan Sun, Chuangui Wu, Qi Yu, Zhen Liu, Tupei Chen, Yang Liu

**Affiliations:** ^1^State Key Laboratory of Electronic Thin Films and Integrated Devices, University of Electronic Science and Technology of China, Chengdu, China; ^2^Beijing China Changfeng Electromechanical Technology Research and Design Institute, Beijing, China; ^3^School of Materials and Energy, Guangdong University of Technology, Guangzhou, China; ^4^School of Electrical and Electronic Engineering, Nanyang Technological University, Singapore, Singapore; ^5^Deepcreatic Technologies Ltd., Chengdu, China

**Keywords:** memristor, working memory, neural networks, bio-inspired computing, Hebbian learning

## Abstract

Working memory refers to the brain's ability to store and manipulate information for a short period. It is disputably considered to rely on two mechanisms: sustained neuronal firing, and “activity-silent” working memory. To develop a highly biologically plausible neuromorphic computing system, it is anticipated to physically realize working memory that corresponds to both of these mechanisms. In this study, we propose a memristor-based neural network to realize the sustained neural firing and activity-silent working memory, which are reflected as dual functional states within memory. Memristor-based synapses and two types of artificial neurons are designed for the Winner-Takes-All learning rule. During the cognitive task, state transformation between the “focused” state and the “unfocused” state of working memory is demonstrated. This work paves the way for further emulating the complex working memory functions with distinct neural activities in our brains.

## 1. Introduction

Working memory is an essential brain function that allows for the temporary storage and manipulation of information required for cognitive tasks (Baddeley and Hitch, 1974; Morris, 1986; Baddeley, 1992, 2010). For a long time, it was thought to be presented in the form of persistent neuronal firing during the delay period (Funahashi, 2017). However, recent studies have suggested that synaptic weight can also store information during the delay period, even if persistent neuronal firing has ceased (Mongillo et al., 2008; Stokes, 2015; Silvanto, 2017). This phenomenon is referred to as “activity-silent” working memory. In most previous studies, the sustained neuronal firing and “activity-silent” working memory have been modeled independently, and these mechanisms appear to be fundamentally opposed in principle. On the other hand, in recent years, several studies have provided insights into the interaction between sustained neuronal firing and “activity-silent” working memory. Manohar et al. have proposed a memory model that unites both persistent activity attractors and silent synaptic memory, which is applicable to many empirical phenomena (Manohar et al., 2019). Barbosa et al. have investigated the interplay between persistent activity and activity-silent dynamics in the prefrontal cortex using monkey and human electrophysiology data (Barbosa et al., 2020).

In the past decades, a great number of efforts had been made for the hardware implementation of a wide variety of artificial neural networks (Misra and Saha, 2010; Capra et al., 2020; Nguyen et al., 2021; Ghimire et al., 2022). Recent works on silent synapses and artificial synapses have highlighted their potential for advancing the understanding of the nervous system and developing neuromorphic computing technologies (Loke et al., 2016; Go et al., 2021; Hao et al., 2021). Following this research trend, there is a growing anticipation to realize a highly bio-plausible neuromorphic computing system. Although working memory plays a vital role in biological neurocomputing (Wang et al., 2020), there have been only a handful of studies on the hardware implementation of working memory, and the existing research has mainly focused on the independent neural mechanism of working memory (Brown and Aggleton, 2001; Ji et al., 2022). Proposing a hardware design for working memory that is compatible with both sustained neuronal firing and activity-silent working memory can improve its biological plausibility and expand the breadth of its application.

To achieve the aforementioned dual functional states of working memory, this paper proposes a hardware design for working memory based on memristors. Memristor is a non-linear two-terminal electrical device that has been extensively studied in the past decade, which is a key element used in artificial neural networks for synapses and neurons due to similarities in electrical behavior (Chua, 1971; Strukov et al., 2008; Thomas, 2013; Li et al., 2018; Camuñas-Mesa et al., 2019; Xia and Yang, 2019). In this work, we propose a memristor-based neural network to realize the dual functional states of working memory. To achieve this, the electrical characteristics of an Au/LNO/Pt memristor based on Single-Crystalline *LiNbO*_3_ (SC-LNO) thin films is utilized. The use of the high-quality SC-LNO thin film results in several advantageous properties, including high switching uniformity, long retention time, stable endurance performance, and reproducible multilevel resistance states (Wang et al., 2022). An artificial synapse circuit with simplified Hebbian learning rule is implemented with Au/LNO/Pt memristor. A spiking neural network capable of realizing the winner-takes-all (WTA) functionality is constructed, which is utilized to achieve working memory working memory. State transformation between the “focused” state (sustained neuronal firing) and the “unfocused” state (activity-silent working memory) of memristor-based working memory is demonstrated. This hardware solution for bio-plausible working memory with dual functional states, leveraging the intrinsic electrical properties of memristors, has promising implications for the development of advanced bio-plausible neuromorphic computing systems.

## 2. Materials and methods

The memristor utilized in this study is an Au/LNO/Pt memristor, which is based on 30 nm Single-Crystalline *LiNbO*_3_ (SC-LNO) thin films. The detailed fabrication method was presented in our prior work (Wang et al., 2022). The electrical characteristics of the memristor were obtained at room temperature using a Keithley 4200-SCS Semiconductor Characterization System. A modified Yakopcic generalized memristor model (Yakopcic et al., 2011) is employed to fit the experimental data of the LNO memristor, taking into account its inherent instability. The dual funtiaonal states of working memory was validated using the Brian spiking neural network simulator (Brian 2). The memristor-based working memory circuit is designed and verified through SPICE simulations.

### 2.1. Dual functional states of working memory

**Figure 1:**
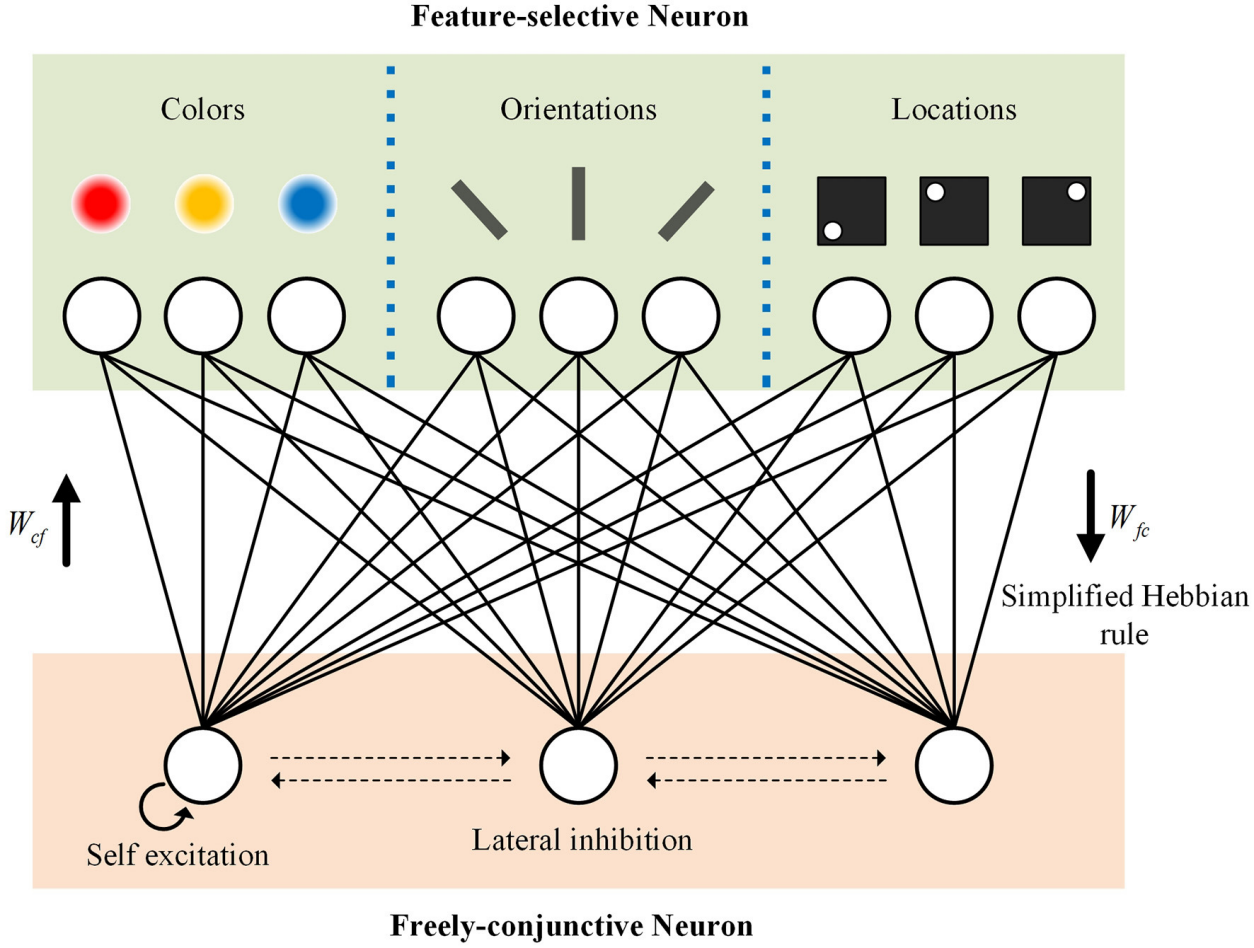
Network architecture of working memory with dual functional states.

Figure 1 depicts the working memory network model that supports “focused” state (sustained neuronal firing) and the “unfocused” state (activity-silent working memory), which builds upon Manohar's working memory model (Manohar et al., 2019) by adjusting it to the form of Spiking Neural Networks (SNNs), thus endowing it with greater biological plausibility. The network comprises two distinct types of neurons: feature-selective neurons and freely-conjunctive neurons. Feature-selective neurons receive unique types of feature information such as colors, orientations, and locations. On the other hand, freely-conjunctive neurons encode a combination of simultaneously active features and establish an associative mapping to feature-selective neurons. Upon arrival of feature information stimulus, the membrane potential of the corresponding feature-selective neuron increases. As the potential of a feature-selective neuron approaches its threshold, the neuron fires a spike. The spike train generated by feature-selective neurons can be interpreted as sensory activation of feature information, and it is perceived by freely-conjunctive neurons. Initially, the synaptic weights between the two types of neurons are randomly assigned, reflecting the connection strength between neurons. As spikes arrive from feature-selective neurons, the membrane potential of freely-conjunctive neurons increases, eventually leading to firing. Synaptic connection strength changes according to the temporal relationship between pre- and post-synaptic spikes, in accordance with the Hebbian plasticity rule. Freely-conjunctive neurons also compete with each other through lateral inhibition and self-excitation, following the winner-takes-all (WTA) rule. Through this competition, only one neuron remains active in each feature dimension. The working memory network model includes two independent weight vectors connecting feature-selective neurons and freely-conjunctive neurons, which depend on the direction of spike propagation. In Figure 1, *W*_*fc*_ indicates the synaptic weight of the forward direction (feature-selective neurons to freely-conjunctive neurons), and *W*_*cf*_ indicates the synaptic weight of the backward direction (freely-conjunctive neurons to feature-selective neurons). During Hebbian learning, both feature-to-conjunctive synapses and conjunctive-to-feature synapses are strengthened for the winner among freely-conjunctive neurons, while they are weakened for other freely-conjunctive neurons. This process creates an synaptic mapping between the freely-conjunctive neurons and the feature information stimulus.

**Figure 2:**
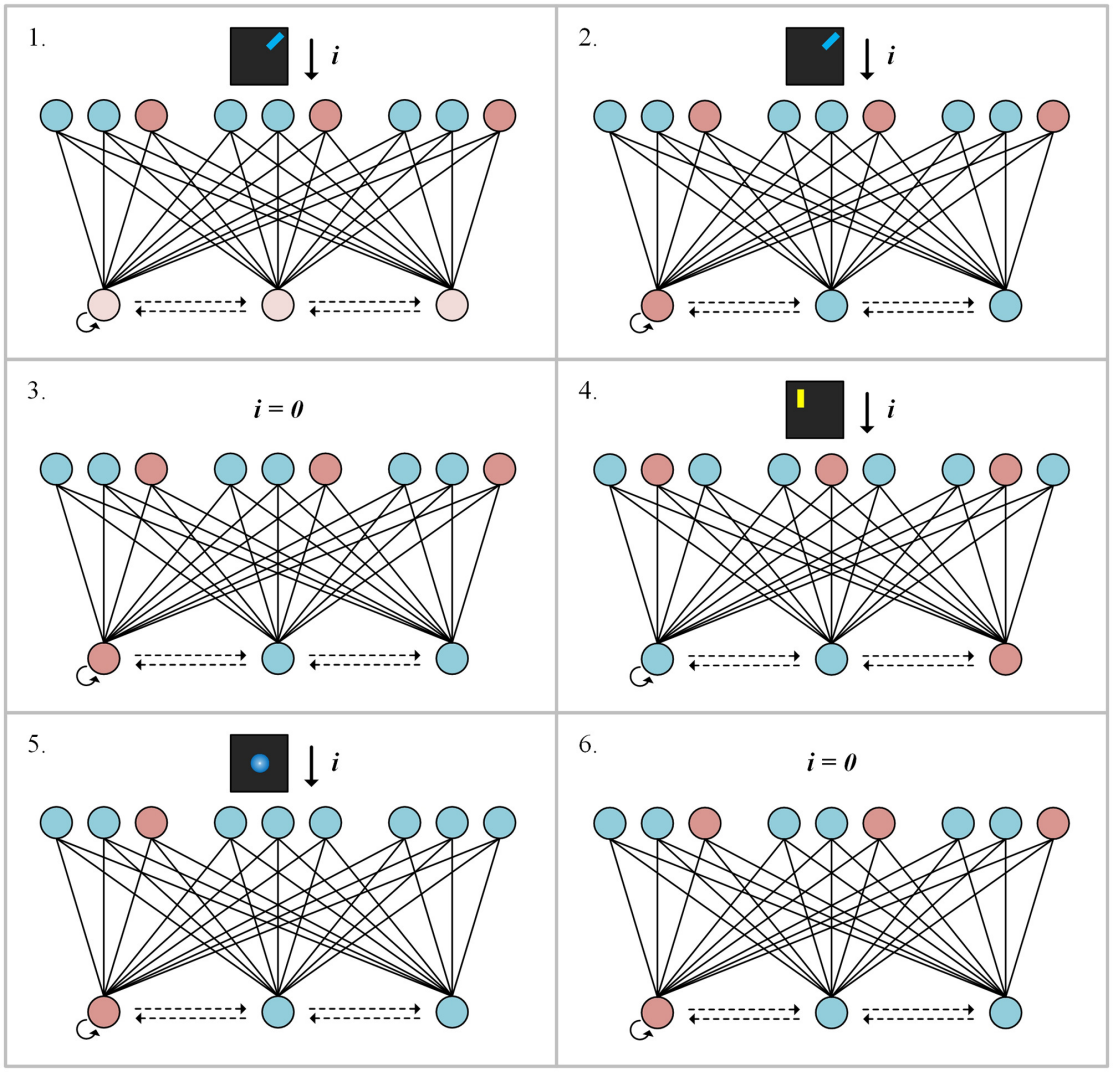
Sequence of neuronal events in working memory.

Figure 2 shows the sequence of neuronal events in working memory: *encoding, attention*, and *retrieval*. Step 1: sensory inputs (color: blue, orientation: +45 degrees, location: top right) are received by feature-selective neurons. Then, freely-conjunctive neurons perceive spike trains from feature-selective neurons. The freely-conjunctive neurons filled with light red indicate relatively lower and erratic firing rates. During this process, the freely-conjunctive neurons compete and encode the active features. Following this, each feature dimension has a single neuron that wins a competition, which can be seen as neuronal events of *encoding* in working memory. Step 2: the neurons filled with deep red indicate higher and more stable firing rates. Under the WTA rule, firing from the winner among the freely-conjunctive neurons is stabilized. Step 3: when the sensory input to the feature-selective neuron is withdrawn, the freely-conjunctive neuron that has been firing stimulates the feature-selective neuron through the conjunctive-to-feature synapses, this results in the firing of the feature-selective neuron and, in turn causes the freely-conjunctive neuron to fire through the feature-to-conjunctive synapses. The self-excitation synapse of the freely-conjunctive neuron enhances its firing through self-stimulation. This sustained neuronal firing can be considered as neuronal events of *attention* in working memory, which indicate “focused” state of working memory. Step 4: a new round of encoding and competition in the working memory network, and different freely-conjunctive neuron from the previous round may enter the “focused” state. Although the feature information from the previous round is not presented in the working memory network in the “focused” state, it remains encoded in synaptic weights as an activity-silent working memory in the “unfocused” state. Step 5: The providing partial feature information to feature-selective neurons, which reactivates the “focused” state of corresponding freely-conjunctive neurons. This can be considered as neuronal events of *retrieval* in working memory. Step 6: freely-conjunctive neuron reactivates original features, completing the process of associative recall. From a certain perspective, the activity-silent working memory in this model can be conceptualized as an associative memory with long-term information storage. Although working memory is typically regarded as a form of short-term memory, several studies suggest that associative memory with long-term information storage also contribute to working memory (Burgess and Hitch, 2005; Olson et al., 2005; van Geldorp et al., 2012). During working memory tasks, as many rounds of sensory inputs are applied, the synaptic mapping of activity-silence working memory may be interrupted by new feature information. This is attributed to the limited capacity of working memory, which is consistent with the concept of *forgetting* in working memory. Although sustained neuronal firing weakens and ceases during the bi-directional feedback between two types of neurons after a relatively short period of time, this process indicates a transition from the “focused” state to the “unfocused” state, rather than *forgetting*.

### 2.2. Synaptic behavior of the LNO memristor

**Figure 3:**
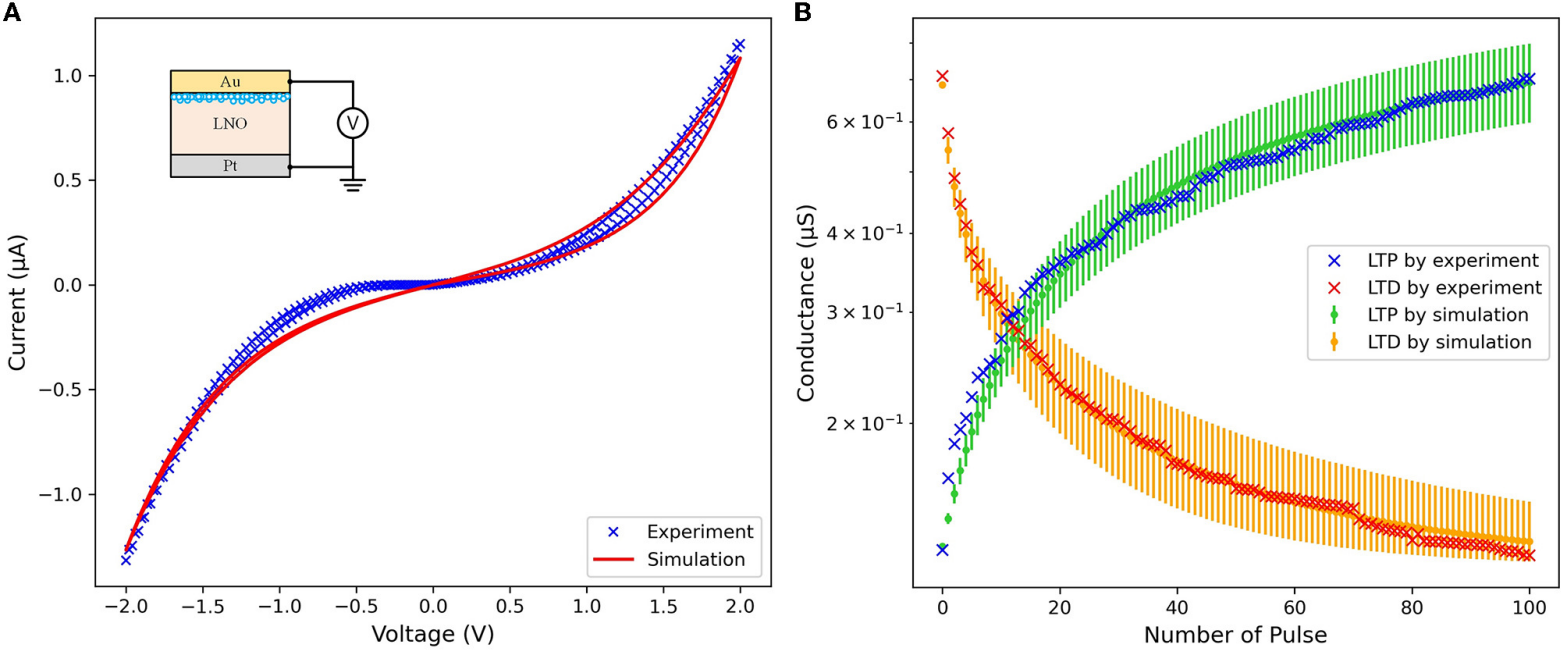
Characteristics of the LNO memristor under **(A)** voltage sweep and **(B)** sequential LTP/LTD pulses.

The cross-sectional structure of the Au/LNO/Pt memristor used in this work is shown in the inset part of Figure 3A. The insulating layer of the memristor is a Single-Crystalline *LiNbO*_3_ (SC-LNO) thin film with the thickness of 30 nm. The cross mark in Figure 3B display the electronic behavior of LNO memristive conductance under appropriate spike trains applied to the memristor. The blue cross mark depict the LTP characteristic of the memristor. Positive pulse trains comprising 100 sequential 1.5 V pulses, each lasting 5ms, are applied to the top electrode (i.e., the Au electrode) of the memristor while the bottom electrode (i.e., the Pt electrode) is grounded, and the memristive conductance gradually increases, indicating that the memristor undergoes a SET process. The red cross mark depict the LTD characteristic of the memristor. Negative pulse trains comprising 100 sequential −1.5 V pulses, each lasting 5 ms, are applied to the bottom electrode (i.e., the Pt electrode) of the memristor while the top electrode (i.e., the Au electrode) is grounded, and the memristive conductance gradually decreases, indicating that the memristor experiences a RESET process.

In this work, a modified Yakopcic's generalized memristor model (Yakopcic et al., 2011) is used to fit the experimental data of the LNO memristor. The equation of the modified memristor model is as follows:


(1) $$\begin{array}{l} I\left(x\right)=\{\begin{array}{c} a_{1}\left(\frac{1}{r}+\frac{\left(r-1\right)x\left(t\right)}{r}\right)\sinh\left(bV\left(t\right)\right),V\left(t\right)\geq 0\\ a_{2}\left(\frac{1}{r}+\frac{\left(r-1\right)x\left(t\right)}{r}\right)\sinh\left(bV\left(t\right)\right),V\left(t\right)<0 \end{array} \end{array}$$

(2) $$\begin{array}{l} g\left(V\left(t\right)\right)=\{\begin{array}{c} A_{p}\left(e^{V\left(t\right)}-e^{V_{p}}\right),V\left(t\right)>V_{p}\\ -A_{n}\left(e^{-V\left(t\right)}-e^{V_{n}}\right),V\left(t\right)<-V_{n}\\ 0,-V_{n}<V\left(t\right)<V_{p} \end{array} \end{array}$$

(3) $$\begin{array}{l} f\left(x\left(t\right)\right)=\{\begin{array}{c} e^{-\alpha_{p}\left(x\left(t\right)-x_{p}\right)}w_{p}\left(x\left(t\right),x_{p}\right),x\left(t\right)\geq x_{p}\\ 1,x\left(t\right)<x_{p} \end{array} \end{array}$$

(4) $$\begin{array}{l} f\left(x\left(t\right)\right)=\{\begin{array}{c} e^{\alpha_{n}\left(x\left(t\right)+x_{n}-1\right)}w_{n}\left(x\left(t\right),x_{n}\right),x\left(t\right)\leq 1-x_{n}\\ 1,x\left(t\right)>1-x_{n} \end{array} \end{array}$$

(5) $$\begin{array}{l} w_{p}\left(x,x_{p}\right)=\frac{x_{p}-x}{1-x_{p}}+1 \end{array}$$

(6) $$\begin{array}{l} w_{n}\left(x,x_{n}\right)=\frac{x}{1-x_{n}} \end{array}$$

(7) $$\begin{array}{l} \frac{dx}{dt}=\eta g\left(V\left(t\right)\right)f\left(x\left(t\right)\right) \end{array}$$

In Equation (1), the hyperbolic sinusoid function is used to fit the I-V relationship of the memristor, along with parameters *a*_1_, *a*_2_, and *b*. The state variable of the memristor is represented by *x*(*t*), which varies between 0 and 1. At *x*(*t*) = 0, the memristor's resistance reaches its maximum value, while *x*(*t*) = 1 corresponds to the minimum resistance. In contrast to the original Yakopcic memristor model, a parameter *r* is introduced in Equation (1) to represent the ratio of the highest resistance value to the lowest resistance value. The purpose of this modification is to adjust the resistance range of the memristor to fall within the range of experimental data and to prevent the occurrence of infinitely large or small resistance values. Equation (2) presents the function *g*(*V*(*t*)), which imposes a programming threshold on the memristor. The positive and negative thresholds are denoted by *V*_*p*_ and *V*_*n*_, respectively, with adjustable parameters for the magnitude of the exponentials represented by *A*_*p*_ and *A*_*n*_. The state variable motion is modeled by the function *f*(*x*(*t*)), which is expressed in Equations (3) and (4). The motion remains constant until the point *x*_*p*_ or *x*_*n*_, where the rate of exponential decay is determined by α_*p*_ or α_*n*_, respectively. The window function *w*_*n*_(*x, x*_*n*_) is utilized to ensure the boundary of the state variable motion, as shown in Equations (5) and (6). Additionally, Equation (7) models the state variable motion, with the direction of the motion represented by η in terms of the voltage polarity. Figure 3A shows the current-voltage (I-V) relationship of the LNO memristor model under a voltage sweep from −2 to 2 V for both experiment and simulation. The LTP and LTD characteristics of the modified Yakopcic's generalized memristor model are illustrated by the green and orange lines in Figure 3B, with the parameters used in this work listed in Table 1. Considering the inherent instability of Cycle-to-cycle and Device-to-device variations in practical memristors, 40, 10, 25, and 20% noise are introduced to α_*p*_, α_*n*_, *A*_*p*_, and *A*_*n*_, respectively. The errorbars on the green and orange lines represent the maximum conductance variations of the memristor after 100 sequential LTD and LTP pulses.

**Table 1:** The parameters of the LNO memristor model.

***a*** **_1_**	***a*** **_2_**	***b***	***r***	***V*** **_*p*_**	***V*** **_*n*_**	***A*** **_*p*_**	***A*** **_*n*_**	***x*** **_*p*_**	***x*** **_*n*_**	**α_*p*_**	**α_*n*_**	***x*** **_*o*_**	**η**
7 × *e*^−7^	7 × *e*^−7^	1.5	9	0.5	0.5	0.95	35	0.05	0.3	1.5	5	0.01	1

### 2.3. Memristor-based working memory circuit

**Figure 4:**
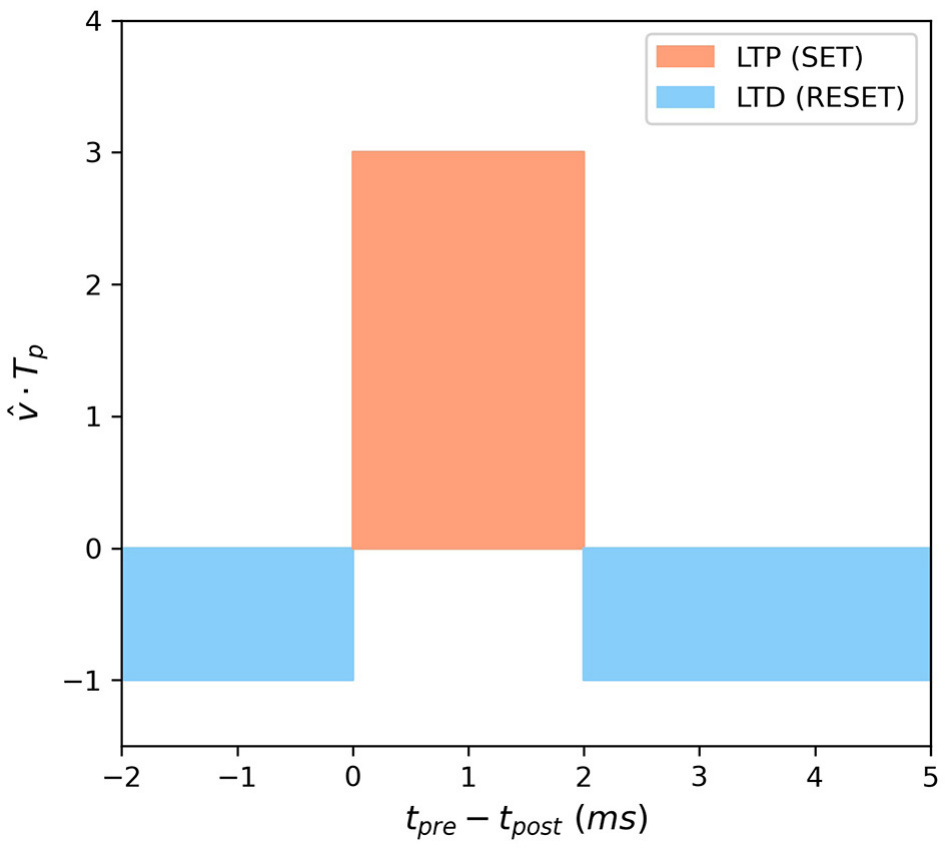
Simplified Hebbian learning rule.

In this study, a simplified Hebbian learning rule is introduced as the synaptic plasticity mechanism for feature-to-conjunctive synapses and conjunctive-to-feature synapses, as illustrated in Figure 4. Long-term potentiation (LTP) and long-term depression (LTD) of synaptic weights are determined by the temporal relation between pre- and post-synaptic spikes. When the pre-synaptic spike is fired first and the post-synaptic spike is fired immediately within a time window of 2ms, the synapse exhibits LTP. When the post-synaptic spike is fired outside of this time window, either earlier or later, the synapse exhibits LTD. The strength of LTP and LTD is determined by the SET/RESET pulse width, denoted by time scalar *T*_*p*_. The pulse width for SET is three times wider than that for RESET. $\hat{v}$ represents the direction of the pulse voltage, with the direction of SET being positive and the direction of RESET being negative.

**Figure 5:**
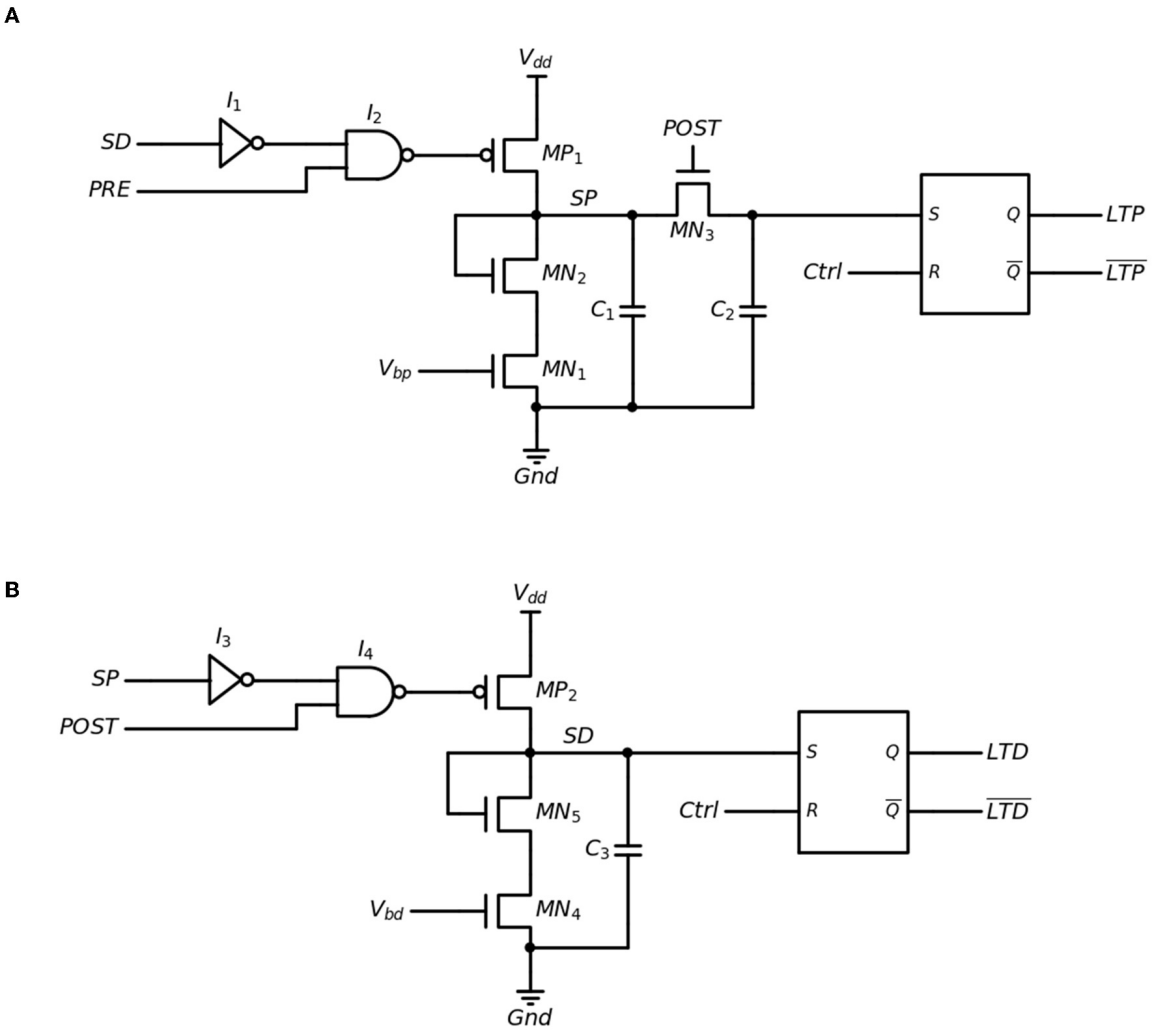
Schematic illustration of **(A)** LTP Module and **(B)** LTD Module.

To harness the LTP/LTD behavior of the LNO memristor, a dedicated synaptic weight update circuit has been designed and optimized based on our previous research (Hu et al., 2013). The synaptic weight update circuit is comprised of a single LTP module, a single LTD module, one memristor, and the peripheral circuit. Schematic representations of the LTP and LTD modules are shown in Figures 5A, B, respectively. The “PRE” and “POST” nodes are connected to the pre-neuron and post-neuron, respectively, while the LTP and LTD nodes represent the output generated by the temporal relation between pre-synaptic and post-synaptic spikes, as specified by the simplified Hebbian learning rule mentioned earlier. At the initial state, both “SP” and “SD” nodes are set to a low level. *I*_1_ and *I*_3_ function as inverters, while *I*_2_ and *I*_4_ function as NAND gates. When the pre-synaptic neuron fires, transistor *MP*_1_ turns on, causing capacitor *C*_1_ to charge up to *V*_*dd*_, and as a result, the “SP” node rises to *V*_*dd*_. The output of *I*_4_ in the LTD module is held at a low level, which means that the LTD module is inactive while the LTP module is operating, and vice versa. When the LTP module is in operation, *C*_1_ begins to discharge through transistors *MN*_1_ and *MN*_2_, with the discharge current being regulated by *V*_*bp*_. The maximum duration for the discharge of *C*_1_ is denoted as *t*_*LTP*_, which represents the time window of the LTP. When the post-synaptic neuron fires within *t*_*LTP*_, transistor *MN*_3_ will be turned on, causing the charge to be redirected to capacitor *C*_2_, and setting the “S” input of the S-R latch to a high level. As a result, the LTP output of the S-R latch will be in a “HIGH” state. When the LTD module is in operation, the “S” input of the S-R latch is immediately set to a high level, causing the LTD output of the S-R latch to be in a “HIGH” state. The only purpose of the discharge of *C*_3_ is to deactivate the LTP module while the LTD module is in operation. The LTP/LTD output will remain in a “HIGH” state until a “Ctrl” signal arrives at the S-R latch, which in turn determines the duration of the LTP/LTD output.

**Figure 6:**
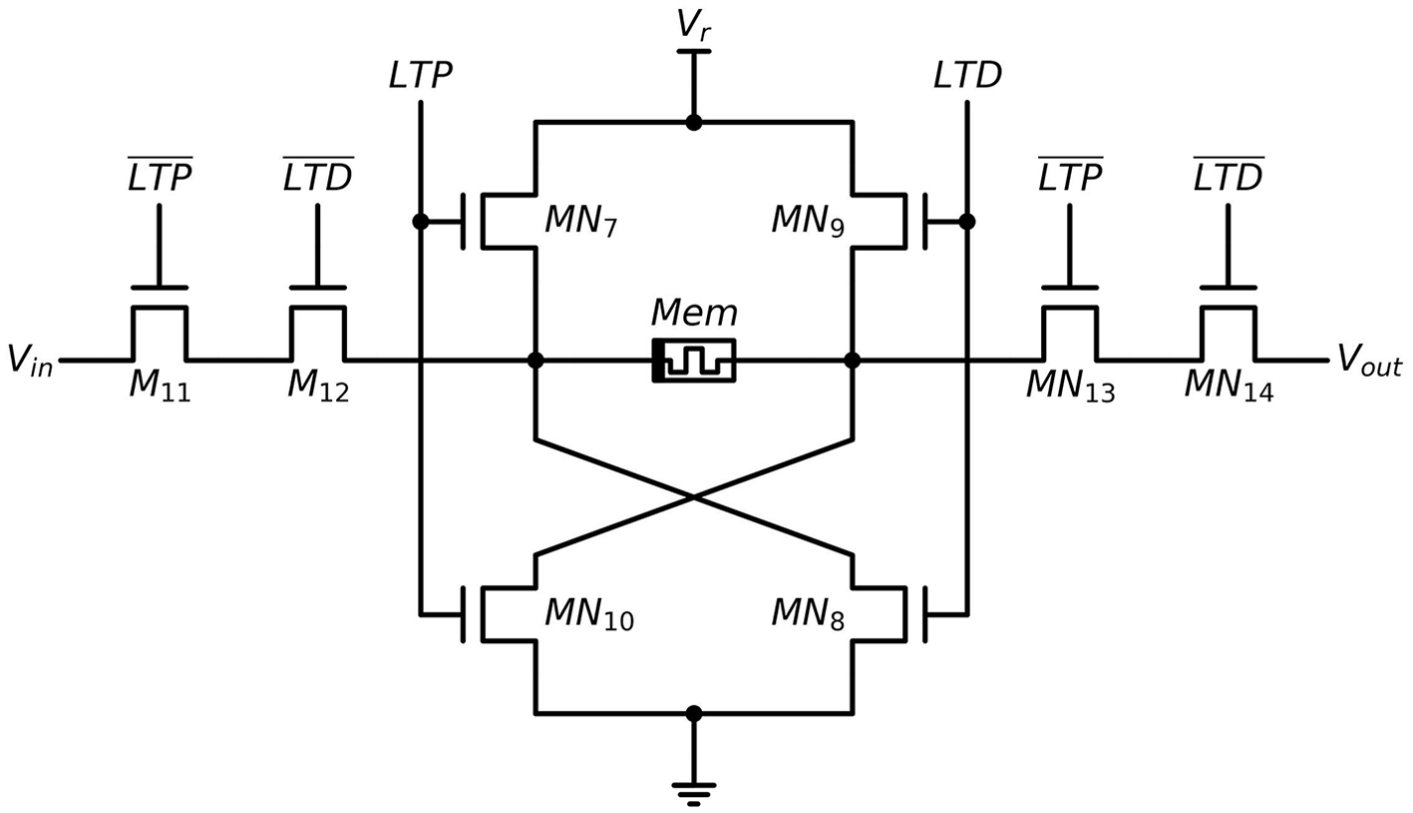
Schematic illustration of synapse weight update circuit.

The LTP and LTD outputs described above are utilized to implement a Hebbian learning rule in the synapse weight update circuit, as depicted in Figure 6. When the LTP output is in a “HIGH” state, transistors *MN*_7_ and *M*_10_ turn on, applying a positive voltage Vr to the memristor with the top electrode at high potential and leading to a SET process in the memristor. Similarly, when the LTD output is in a “HIGH” state, transistors *MN*_8_ and *MN*_9_ turn on, applying *V*_*r*_ to the memristor with the bottom electrode at low potential, leading to a RESET process in the memristor. The duration of the SET/RESET process is equal to the duration of the LTP/LTD output. When the LTP and LTD outputs are in a low state, their inverted outputs $\bar{LTP}$ and $\bar{LTD}$ are in a high state. As a result, transistors *MN*_11_-*MN*_14_ in the path between the pre-synaptic neuron and the post-synaptic neuron remain on, allowing pre-synaptic spikes to transmit to the post-synaptic neuron through the memristor. However, when the LTP or LTD module is activated, two of the transistors among *MN*_11_-*MN*_14_ turn off, and the path between the pre-synaptic neuron and the post-synaptic neuron is closed.

To achieve working memory, we utilized a spiking neural network with the winner-takes-all (WTA) functionality previously developed in our research (Wang et al., 2019). All synapses involved in working memory, including feature-to-conjunctive synapses, conjunctive-to-feature synapses, self-exciting synapses, and lateral-inhibition synapses, share the same synaptic architecture. The synapse weight of feature-to-conjunctive and conjunctive-to-feature synapses can be modified by the aforementioned synapse weight update circuit that utilizes a simplified Hebbian learning rule. On the other hand, the synaptic weight of self-exciting synapses and lateral-inhibition synapses remain fixed. Both feature-selective neurons and freely-conjunctive neurons in our network utilize the leaky integrate-and-fire (LIF) neuron model. The capacitor within the neuron integrates the input current from the synapses, causing the neuron's membrane potential to increase. Once the neuron potential reaches the threshold, the neuron fires a spike, and its potential returns to its resting state. The network topology of our working memory design, consisting of interconnected artificial neurons and synapses as illustrated in Figure 1.

## 3. Results and discussion

The functionality of the memristor-based working memory was evaluated using SPICE simulation, leveraging the electrical characteristics of Au/LNO/Pt memristors derived from experimental data. The memristive conductance was normalized to serve as synaptic weights. The working memory employed a total of 9 feature-selective neurons, with each group of 3 neurons corresponding to a distinct feature dimension, including color, orientation, and location. Each neuron was responsible for encoding different feature information within its respective dimension. Additionally, 3 freely-conjunctive neurons were fully-connected to the feature-selective neurons, resulting in a total of 27 feature-to-conjunctive and 27 conjunctive-to-feature synapses. To enable lateral inhibition, 6 synapses were established between freely-conjunctive neurons, with self-connections excluded. Lastly, 3 self-exciting synapses were connected to the freely-conjunctive network in a self-connected manner, for the purpose of inducing self-excitation.

**Figure 7:**
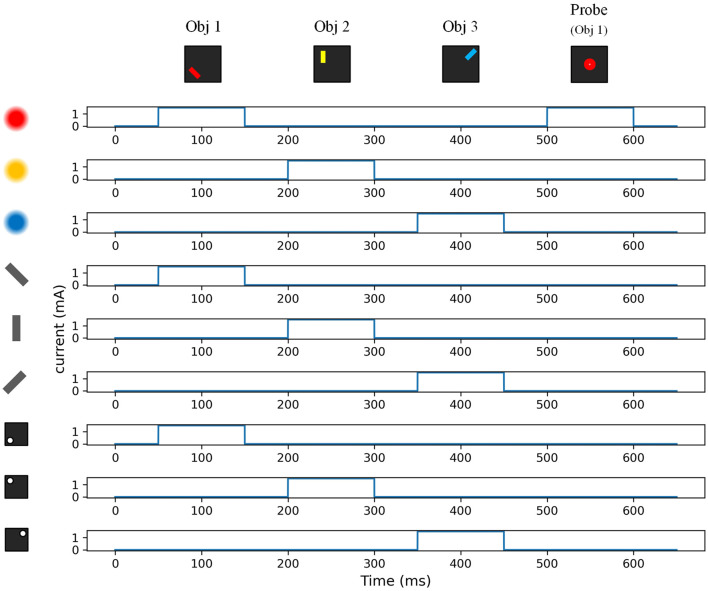
Sensory input of feature-selective neurons.

Figure 7 shows a typical sensory input for working memory. Three input features were selected: *Obj*_1_ (color: red, orientation: −45 degrees, location: bottom-left), *Obj*_2_ (color: yellow, orientation: 0 degrees, location: top-left), and *Obj*_3_ (color: blue, orientation: +45 degrees, location: top-right). The sensory current was applied to corresponding feature-selective neurons, and the magnitude of the sensory current was adjusted to the same value for each of the 3 feature dimensions. The firing rate of the feature-selective neuron was proportional to the magnitude of the input current. An initialization time of 50 ms was allocated for stabilization at the beginning of working memory. Each feature input lasted for 100 ms, and there was a 50 ms resting time between each two features. The time scalar *T*_*p*_ for the simplified Hebbian learning rule is defined as 1 ms.

**Figure 8:**
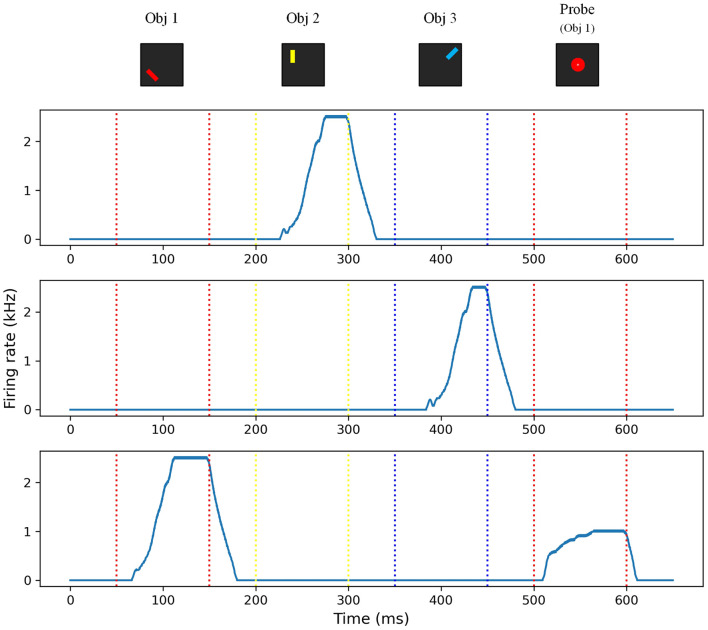
Firing rate of freely-conjunctive neuron.

Figure 8 shows the activity of each freely-conjunctive neuron. When the first feature input *Obj*_1_ is activated, feature-to-conjunctive synapses modify their connectivity based on a simplified Hebbian learning rule, and freely-conjunctive neurons compete with each other. This process is known as *encoding* in working memory. Once the competition is completed, the winning neuron remains active even without the sensory input. The feature information is encoded into working memory and is presented in the form of persistent neuronal firing, which can be regarded as the “focus” state of working memory. Additionally, feature information is silently encoded into the synaptic weights of the feature-to-conjunctive synapses. When the second feature *Obj*_2_ is activated, the previous *attention* is disturbed by the new input, and a new round of *encoding* occurs. Then, the neurons that won the competition in this round persistently fire, forming a new *attention*. Similarly, when the third feature input *Obj*_3_ is activated, another “focus” state of working memory is formed as the persistent firing freely-conjunctive neurons map to *Obj*_3_. However, the “unfocused” state of working memory exists in the form of synaptic mappings and persists as long as it is not overwritten by new feature information during the working memory task. After a resting time of 50 ms, a sensory input composed of partial feature information of *Obj*_1_ (colors: red) is applied. Despite using only a small fraction of the feature information, persistent neuronal firing occurs again, which corresponds to the retrieval of *Obj*_1_. This result demonstrates that the proposed memristor-based working memory system can successfully achieve the dual functional states of working memory and accomplish the working memory task.

## 4. Conclusion

In this paper, a memristor-based working memory that is capable of exhibiting dual functional states is presented. To achieve this, an artificial synapse with a simplified Hebbian learning rule was designed based on the LTP/LTD properties of the Au/LNO/Pt memristor, which uses a single-crystalline LiNbO3 (SC-LNO) thin film as its insulating layer. Two types of artificial LIF neurons were implemented in the network to encode feature information to working memory through the WTA rule and produce persistent neuronal firing patterns. The results show that the proposed system can realize various neuronal events in working memory, including encoding, attention, and retrieval. This study demonstrates that the memristor-based working memory can exist in the dual functional states: the sustained neuronal firing and activity-silent working memory. This study paves the way for the development of advanced bio-plausible neuromorphic computing systems based on memristive neural networks. This research represents a significant step toward the development of advanced bio-plausible neuromorphic computing systems based on memristive neural networks. It is hoped that this work will inspire further research in this exciting and rapidly evolving field.

## Data availability statement

The raw data supporting the conclusions of this article will be made available by the authors, without undue reservation.

## Author contributions

HW and JW conceived of the presented idea, designed the circuits and performed the computations and simulations, and prepared the manuscript with contributions from all authors. HW, XP, and JW carried out the experiment. XP and CW fabricated the device. HW and XP analyzed the data. MS, QY, and ZL verified the analytical methods. YL, CW, and TC were in charge of overall direction and planning. All authors contributed to the article and approved the submitted version.
